# A case of endogenous endophthalmitis caused by Escherichia coli septicemia

**DOI:** 10.3205/oc000239

**Published:** 2024-06-25

**Authors:** Tejinder Talwar, Prateek Chandra, Sugandha Goel, Kuntal Patel

**Affiliations:** 1 Department of Medicine, Maharishi Markandeshwar Institute of Medical Sciences and Research, Mullana, Haryana, India; 2 Department of Ophthalmology, Maharishi Markandeshwar Institute of Medical Sciences and Research, Mullana, Haryana, India

**Keywords:** endophthalmitis, endogenous endophthalmitis, pyelonephritis, E. coli septicemia

## Abstract

Endogenous endophthalmitis is a severe sight-threatening condition that requires urgent intervention. It is a rare complication of *Escherichia coli* septicemia. We herein report a case of left eye endogenous endophthalmitis with uncontrolled type 2 diabetes mellitus with pyelonephritis associated with *Escherichia coli* septicemia. Vitrectomy was done along with intravitreal antibiotics and steroids. There was significant improvement in vision after vitrectomy.

## Introduction

Endogenous endophthalmitis (EE) is the intraocular infection affecting the inner coats of the eye with progressive vitreous inflammation that occur as a result of intraocular colonization of microorganisms through hematogenous route [1]. It can lead to permanent loss of vision if not treated on time. *Escherichia coli (E. coli)* induced septicemia can rarely cause endophthalmitis and only few cases have been reported in literature. Herein we report the case of a patient with acute pyelonephritis associated with *E. coli* septicemia that developed EE during his hospital course.

## Case description

**Figure 1:**
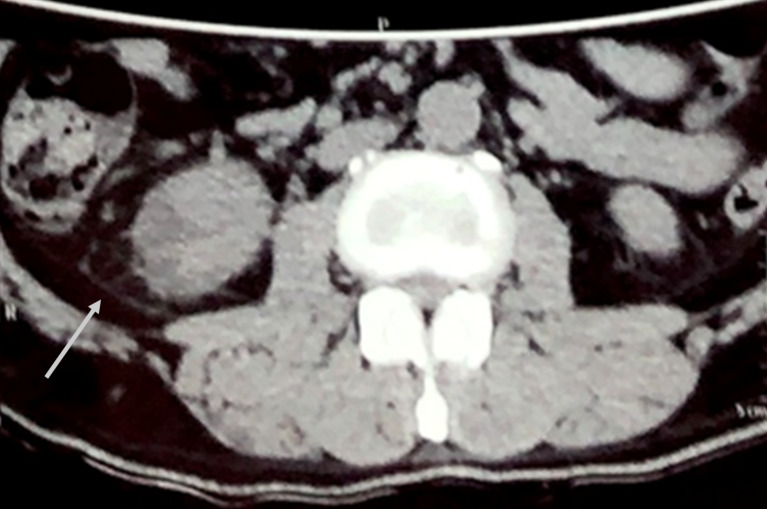
NCCT abdomen showing fuzzy cortical margins with perinephric fat stranding suggestive of pyelonephritis

**Figure 2:**
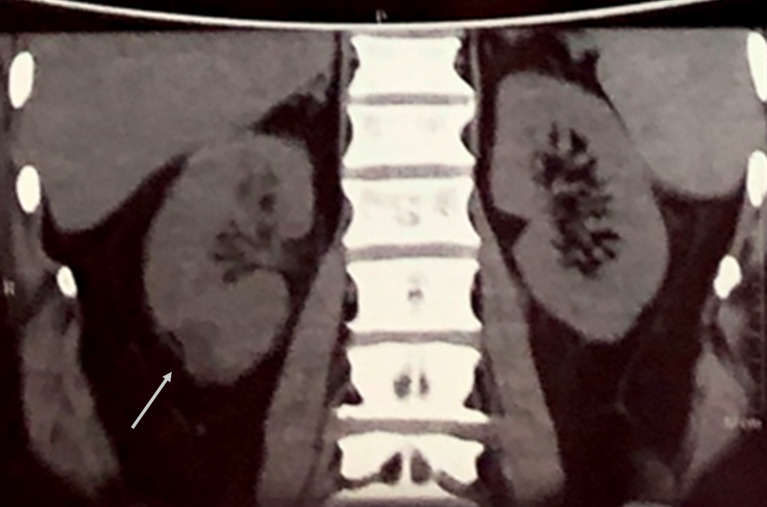
NCCT abdomen showing irregular hypodense area seen in the cortex of lower pole of right kidney

A 55-year-old poorly controlled diabetic male patient presented to our hospital with a history of high grade fever, pain in right flank radiating to back associated with burning micturition for 3 days. He was a known diabetic for 1 year and was taking oral hypoglycemic drugs. On admission, pulse rate was 110/min, blood pressure was 100/80 mmHg, respiratory rate was 20 per min and temperature was 39°C. His ophthalmic, cardiovascular system, respiratory system and central nervous system examination were within normal limits. His abdominal examination revealed tenderness over right renal angle. Blood sugar fasting was 294 mg% and HbA1c was 9.7%. Urine examination revealed no albumin or sugar, pus cells were present on microscopy. Renal function test showed blood urea – 114 mg/dl, serum creatinine – 2.85 mg%, sodium – 132 mEq/L, potassium – 3.9 mEq/L. Urine culture showed no growth. Blood culture showed growth of *E. coli*. Non-contrast computerized tomography (NCCT – abdomen) showed fuzzy cortical margins with perinephric fat stranding and focal hypodense area of 2.3 cm diameter in the cortex of the lower pole of the right kidney suggestive of pyelonephritis (Figure 1 (Fig. 1) and Figure 2 (Fig. 2)). No evidence of renal calculi or hydronephrosis was noted. The diagnosis of acute pyelonephritis with *E. coli* septicemia was made and the patient was treated with intravenous vancomycin, amikacin and tazobactam. He had an episode of confusion with worsening of his renal parameters. His serum urea increased to 220 mg/dl and creatinine increased to 3.5 mg%. Hyperkalemia was noted (potassium – 6.1 mEq/l). Acute kidney injury was suspected and managed conservatively. After 3–4 days, the patient improved significantly and he became afebrile. 

**Figure 3:**
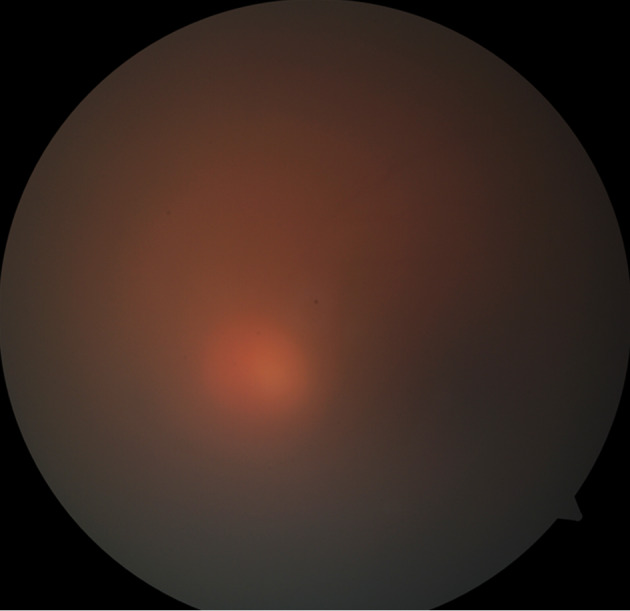
Hazy view of fundus with vitreous exudates

**Figure 4:**
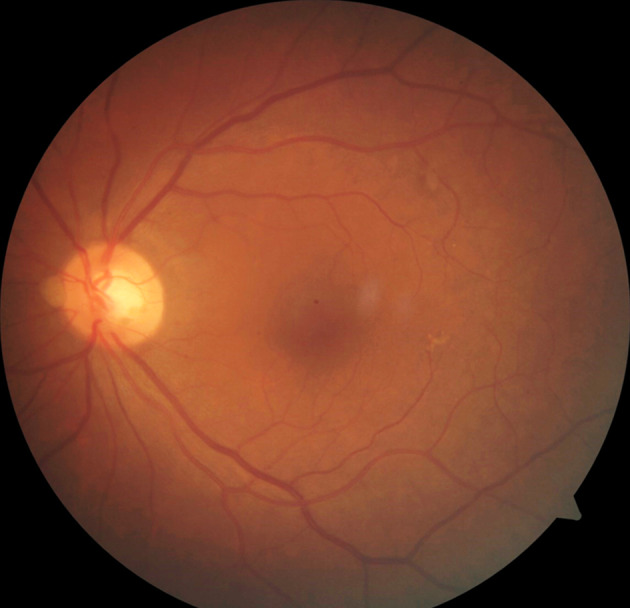
Clear view of fundus post vitrectomy

On the 8^th^ day of admission, the patient complained of a sudden painful diminution of vision in the left eye. Ophthalmic examination was done. His best corrected visual acuity was 6/6 in the right eye and hand movements in the left eye. The right eye was within normal limits. Anterior segment examination of the left eye showed lid edema, circumciliary congestion with cells 4+ and flare 3+ with hypopyon. Fundus showed vitreous haze 4+ and vitreous exudates (Figure 3 (Fig. 3)). Retinal details were not clinically visible. B scan ultrasonography showed plenty of low to moderate reflective dot echoes and few membrane echoes in vitreous. The retina was attached throughout. The patient was diagnosed with EE in left eye. The patient underwent vitrectomy with intravitreal vancomycin (1 mg/0.05 ml), ceftazidime (2.25 mg/0.05 ml) and dexamethasone (0.4 mg/0.05 ml) (Figure 4 (Fig. 4)). Vitreous biopsy was sent for microscopy, culture and sensitivity. Vitreous culture showed no growth. Subsequently, intravitreal injections of antibiotics and steroids were repeated three times at an interval of 2 days. Vision improved to 6/9 postoperatively on 2 weeks follow up. 

## Discussion

Endophthalmitis is a rare and severe disease with the potential to cause visual loss. EE results from hematogenous spread of infective agents from distant infective foci. The most common risk factors associated with EE include diabetes, HIV infection, intravenous drug use, catheterization, renal failure requiring dialysis, chronic liver diseases, cardiac disease, long-term use of broad-spectrum antibiotics, steroids and other immunosuppressive states (which comprises patients with malignancies, post-transplant and on immunosuppressive drugs/chemotherapy) [1]. EE represents 2–17% of all cases of infectious endophthalmitis [2], [3]. Both bacteria and fungi have been reported as etiological agents for EE. In a study of EE conducted in India by Ratra et al., gram-negative bacteria were found to be more common than fungi [4]. Most cases of EE in East Asia are caused by gram-negative organisms, especially *Klebsiella* species accounting for 80 to 90% of positive culture [5].

There are extensive geographical variations among the microbial that cause EE. Fungi are predominant especially in western world, whereas in East Asia, it is gram-negative bacteria. In one study from South India, fungi and bacteria were evenly distributed, *Aspergillus fumigatus* and *Staphylococcus aureus* being the most commonly isolated organisms, respectively. Fungal EE was more commonly seen in immunosuppressed state and bilateral cases [6].

*E. coli* septicemia usually follows urinary tract infections. It occurs frequently in debilitated elderly people with diabetes mellitus. In a study by Pillai et al., diabetes mellitus was commonest predisposing factor (70.7%) for EE [6]. They found out urinary tract infection (32%) was the most common source followed by sepsis (20.5%), abscess (14%) and infective endocarditis (5.8%) [6].

Shammas et al. noted that suppression of the normal bacterial flora by broad spectrum antibiotics has led to the emergence and proliferation of the more resistant gram-negative bacteria [7]. Our patient was a poorly controlled diabetic and developed acute pyelonephritis in his right kidney, which led to septicemia. This septicemia led to metastatic embolization to his eye leading to EE. Such cases of EE occur when pathogens enter the systemic circulation and cross the blood–retina barrier, thereby infecting the ocular tissue. 

Our patient developed visual symptoms on 8^th^ day of admission, which were acute in nature. The interval between onset of systemic symptoms and onset of ocular symptoms was studied by Muda et al. who noted the interval was less than one week in 26% cases, between one to two weeks in 24% cases, 2 weeks to one month in 11% cases and more than one month in 37% of his cases [5]. The onset of visual loss is acute in most bacterial EE as compared to sub-acute onset in fungal EE and prognosis is poor in fungal EE.

Blood culture in our patient showed growth of gram-negative bacteria *E. coli*, while the urine culture and vitreous culture was sterile. Muda et al. noted that most common systemic risk factor was diabetes mellitus and found positive culture from ocular fluid or other body fluids in almost 69% of samples. Blood culture had the highest positive source in 42% cases. It showed growth of gram-negative bacteria in 52%, gram positive bacteria in 40% and fungi in 8% cases. They noted vitreous culture was positive in only 22% cases [5]. Prognosis of EE is often poor. Delay in appropriate diagnosis and treatment can lead to poor visual outcome. A case has been reported in which a patient progressed to panophthalmitis following *E. coli* sepsis requiring evisceration [8].

## Conclusion

In general EE is considered an emergency, which runs a potentially devastating course leaving very limited visual function in many patients. There is a possibility that vision-threatening complication such as endophthalmitis is overlooked by the treating physician as conjunctivitis. Timely intervention is important to save vision. In our patient vision improved significantly to 6/9 after 2 weeks following vitrectomy and intravitreal antibiotics. So, once there is suspicion of EE, early diagnosis and aggressive treatment with targeted antimicrobial therapy are fundamental to improve the outcome.

## Notes

### Competing interests

The authors declare that they have no competing interests.
